# Supplementary material for: A Comprehensive Study on the Nutritional Profile and Shelf Life of a Custom-Formulated Protein Bar Versus a Market-Standard Product
Source: Foods. 2025 Jun 19;14(12):2141. doi: 10.3390/foods14122141 (PMC12191461; doi:10.3390/foods14122141)
Supplement: Supplementary file 1 [file foods-14-02141-s001.zip › foods-3590272-supplementary.pdf]

Supplementary Table S1 – Ingredient List of the Commercial Protein Bar

Ingredient	Description/Function
Soy protein isolate	Primary protein source
Tapioca starch	Carbohydrate and texture enhancer
Palm oil	Fat source
Fructose syrup	Sweetener
Cocoa powder	Flavoring
Glycerin	Humectant for moisture retention
Salt	Flavor enhancer
Emulsifier (soy lecithin)	Stabilizer and emulsifier
Natural flavors	Flavor enhancer
Preservatives	Shelf life extender (exact type not disclosed)
